# Supplementary material for: Recruitment in Health Services Research—A Study on Facilitators and Barriers for the Recruitment of Community-Based Healthcare Providers
Source: Int J Environ Res Public Health. 2021 Oct 7;18(19):10521. doi: 10.3390/ijerph181910521 (PMC8508262; doi:10.3390/ijerph181910521)
Supplement: Supplementary file 1 [file ijerph-18-10521-s001.zip › Table S2_ Topics of the Interviewguide.pdf]

International Journal of
Environmental Research and Public Health

MDPI

Topics of the Interviewguide

**Topic**	**Question**
Icebreaker question	Please tell me about how you were involved in recruiting health care providers.
Motivation for participation in the trial	What do you say was the reason for health care providers to participate in the project?
Refusal to participate in the trial	What reasons for refusal to participate did health care providers communicate to you?
	In your opinion, are there any other reasons why health care providers did not want to participate which they did not explicitly express?
Recruitment strategy	What was the most difficult aspect in recruiting health care providers to the trial?
	Which recruitment strategies have been most successful in your opinion?
Recruitment of patients	Are there commonalities among actively recruiting physician practices?
	Which barriers for recruitment of patients did health care providers report to you?
Ideas for optimization	What would you do differently if you were planning to recruit for a similar trial?
