# Supplementary material for: Recruitment in Health Services Research—A Study on Facilitators and Barriers for the Recruitment of Community-Based Healthcare Providers
Source: Int J Environ Res Public Health. 2021 Oct 7;18(19):10521. doi: 10.3390/ijerph181910521 (PMC8508262; doi:10.3390/ijerph181910521)
Supplement: Supplementary file 1 [file ijerph-18-10521-s001.zip › Table S1_ Data base_documentary analysis.pdf]

International Journal of
Environmental Research and Public Health

MDPI

Additional File 1: Data base for the documentary analysis

**Document type**	**Number of available documents**	**Number of documents included in the analysis**
Records of trial staff meetings and telephone conferences	21	19
Records of health care provider training-sessions	23	12
Records of information events, quality circles and meetings with occupational associations to inform about the trial	17	14
Records of recruitment planning meetings	7	7
Records of regular meetings between study coordinators and research team	30	27
Feedback of enrolled health care providers	26	10
Recruitment material (flyer, letters)	2	0
Records of study coordinator meetings	11	10
**Summe**	137	99

* These are process-produced data that were not created solely for the purpose of scientific evaluation. Various participants contributed to the creation of the records. After the records were created, they were circulated among the team, thus generating an internal consensus within the project.
